# Amazon Rainforest Exchange of Carbon and Subcanopy Air Flow: Manaus LBA Site—A Complex Terrain Condition 

**DOI:** 10.1100/2012/165067

**Published:** 2012-04-24

**Authors:** Julio Tóta, David Roy Fitzjarrald, Maria A. F. da Silva Dias

**Affiliations:** ^1^Universidade do Estado do Amazonas, Manaus, AM, Brazil; ^2^State University of New York, Albany, NY, USA; ^3^Universidade de São Paulo, Sao Paulo, SP, Brazil

## Abstract

On the moderately complex terrain covered by dense tropical Amazon Rainforest (Reserva Biologica do Cuieiras—ZF2—02°36′17.1′′ S, 60°12′24.4′′ W), subcanopy horizontal and vertical gradients of the air temperature, CO_2_ concentration and wind field were measured for the dry and wet periods in 2006. We tested the hypothesis that horizontal drainage flow over this study area is significant and can affect the interpretation of the high carbon uptake rates reported by previous works at this site. A similar experimental design as the one by *Tóta et al.* (2008) was used with a network of wind, air temperature, and CO_2_ sensors above and below the forest canopy. A persistent and systematic subcanopy nighttime upslope (positive buoyancy) and daytime downslope (negative buoyancy) flow pattern on a moderately inclined slope (12%) was observed. The microcirculations observed above the canopy (38 m) over the sloping area during nighttime presents a downward motion indicating vertical convergence and correspondent horizontal divergence toward the valley area. During the daytime an inverse pattern was observed. The micro-circulations above the canopy were driven mainly by buoyancy balancing the pressure gradient forces. In the subcanopy space the microcirculations were also driven by the same physical mechanisms but probably with the stress forcing contribution. The results also indicated that the horizontal and vertical scalar gradients (e.g., CO_2_) were modulated by these micro-circulations above and below the canopy, suggesting that estimates of advection using previous experimental approaches are not appropriate due to the tridimensional nature of the vertical and horizontal transport locally. This work also indicates that carbon budget from tower-based measurement is not enough to close the system, and one needs to include horizontal and vertical advection transport of CO_2_ into those estimates.

## 1. Introduction

The terrestrial biosphere is an important component of the global carbon system. The long-term exchanges estimate of terrestrial biosphere is a challenge and has resulted in ongoing debate [1, 2]. For monitoring long-term net ecosystem exchange (NEE) of carbon dioxide, energy, and water in terrestrial ecosystems, tower-based eddy covariance (EC) techniques have been established worldwide [1].

It is now recognized that the EC technique has serious restrictions for application over complex terrain and under calm and stable nighttime conditions with low turbulence or limited turbulent mixing of air [2–8]. To overcome this problem, the friction velocity (u*)-filtering approach has been formalized by the FLUXNET committee for the estimation of annual carbon balances [5, 9]. This approach simply discarded calm night's flux data (often an appreciable fraction of all nights) and replaced them with ecosystem respiration rates found on windy nights [10]. Papale et al. [11] pointed out that this approach itself must be applied with caution and the friction velocity (u*) corrections threshold is subject to considerable concerns and is very site specific. Miller et al. [10] reported that depending on the u* threshold value used to correct the flux tower data at Santarem LBA site (Easterly Amazon Region—Brazil), the area can change from carbon sink to neutral or carbon source to the atmosphere. 

The transport of CO_2_ by advection process has been suggested by several studies as the principle reason for the “missing” CO_2_ at night [12–17]. The search for this missing CO_2_ has spurred a great deal of research with the goal of explicitly estimating advective fluxes in field experiments during the last decade, in order to correct the NEE bias over single tower eddy covariance measurements [8, 15–25]. 

The complexity of topography and the presence of the valley close to the eddy flux tower have increased the importance to investigating if subcanopy drainage flow account for the underestimation of CO_2_ respiration as past studies have asserted [26]. The Manaus LBA site (Central Amazon Region—Brazil) is an example of moderately complex terrain covered by dense tropical forest. The NEE bias is reported by preview works [27, 28], and a possible explanation for this is that advection process is happening in that site. This work examines subcanopy flow dynamics and local microcirculation features and how they relate to spatial and temporal distribution of CO_2_ on the Manaus LBA Project site. The contribution of exchange of carbon between the atmosphere and the tropical Amazon Rainforest is discussed and correlated with the present work.

## 2. Material and Methods

### 2.1. Site Description

**Figure 1:**
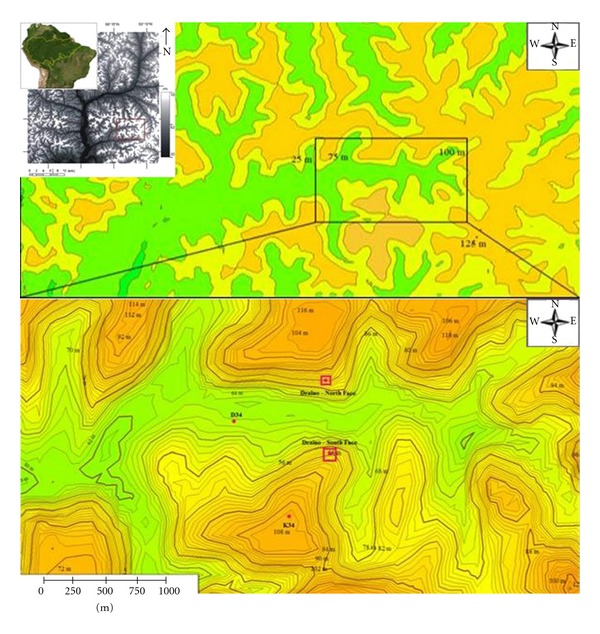
Detailed measurements shows towers' view in the ZF-2 Açu catchment (East-West valley orientation) from SRTM-DEM datasets. The large view in the above panel and below panel: the points of measurements (B34-Valley, K34-Plateau, and subcanopy DRAINO system measurements over slopes in south and north faces (red square).

**Figure 2:**
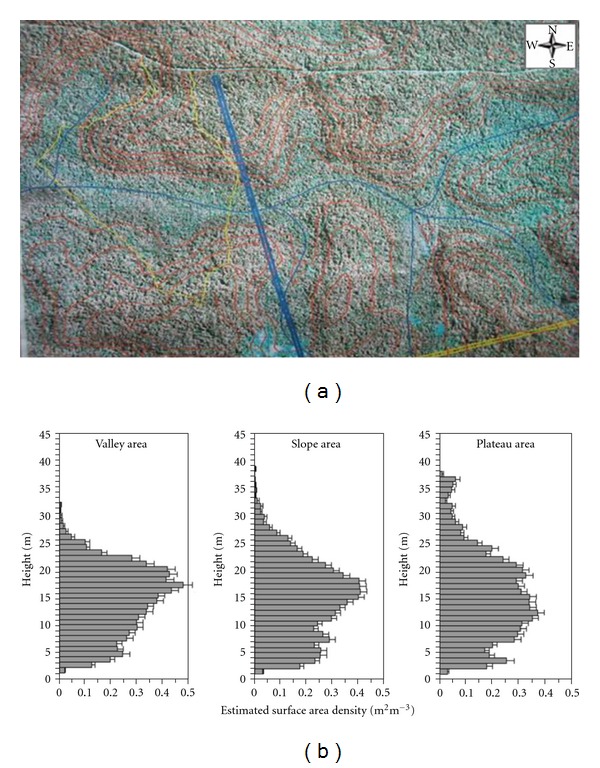
(a) IKONOS's image of the site at the Açu catchment with level terrain cotes and vegetation cover and (b) vegetation structure measured from LIDAR sensor over yellow transect in (a). From (a) the valley vegetation (blue color) and vegetation transition to plateau areas (red colors).

The study site is located in the Cuieiras Biological Reserve (54° 58′W, 2° 51′S), controlled by National Institute for Amazon Research (INPA), about 100 km northeast from the Manaus city. At this site, named K34, was implemented a flux tower with 65 m height to monitor long term microclimate, energy, water, and carbon exchanges [29], and various studies have been conducted in its vicinity. The measurements are part of the Large-Scale Biosphere-Atmosphere experiment in Amazonia.  Figure 1 presents the study site location including the topographical patterns where the maximum elevation is 120 m and the total area (upper panel) is 97.26 km^2^, with distribution of the 31% of plateau, 26% of slope, and 43% of valley [30]. The site area is formed by a topographical feature with moderately complex terrain including a landscape with mosaics of plateau, valley and slopes, with elevation differences about 50 m (Figure 1) and with distinct vegetation cover (Figure 2). The eddy flux tower at the Manaus K34 site has footprints that encompass this plateau-valley mosaic.

The vegetation cover on the plateau and slope areas is composed by tall and dense terra firme (nonflood) tropical forest with height varying between 30 to 40 m, maximum surface area density of 0.35 m^2^ m^−3^ (Figure 2(b), see also [31], and average biomass of 215 and 492 ton.ha^−1^ [32, 33]). 

On the valley area, the vegetation is open and smaller with heights from 15 to 25 m, but with significant surface area density more than the 0.35 m^2^ m^−3^ (Figure 2(b)). The soil type on the plateau and slopes area is mainly formed by oxisols (USDA taxonomy) or clay-rich ferralsols ultisols (FAO soil taxonomy), while, on the valley area, waterlogged podzols (FAO)/spodosols (USDA) with sand soil low drained predominate. Also, in the valley area the presence of small patchy of *campinarana* typical open vegetation with low biomass is also common [34].

The precipitation regime on the site shows wet (December to April) and dry (June to September—less than 100 mm·month^−1^) periods. The total annual rainfall is about 2400 mm and the average daily temperature is from 26 (April) to 28°C (September). For more detailed information about the meteorology and hydrology of this site, see Waterloo et al. [35] and Cuartas et al. [36].

### 2.2. Measurements and Instrumentation

The datasets used in this study include a measurement system to monitor airflow above and below the forest, horizontal gradients of CO_2_, and the thermal structure of the air below the canopy, named “DRAINO System” (see [8]). The data used in this study were collected during the wet season (DOY 1–151) and the dry season (DOY 152–250) of the year 2006. Complementary information was used from flux tower K34 (LBA tower) on the plateau, and sonic anemometer data collected in the valley flux tower (B34; see [28] for details). The flux tower K34 includes turbulent EC flux and meteorological observations of the vertical profiles of the air temperature, humidity and CO_2_/H_2_O concentrations, and vertical profile of wind speed, as well as radiation measurements. The fast-response eddy flux data were sampled at 10 Hz and slow response (air temperature and wind profiles) at 30 min average (see [29] for detailed information).

**DRAINO Measurement System: Manaus LBA ZF2 site**

**Figure 3:**
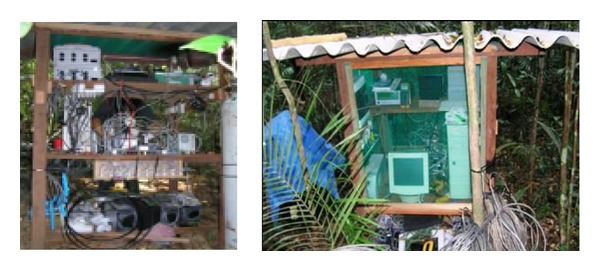
DRAINO measurement system used in Manaus LBA (south face; see also Figure 4).

**Figure 4:**
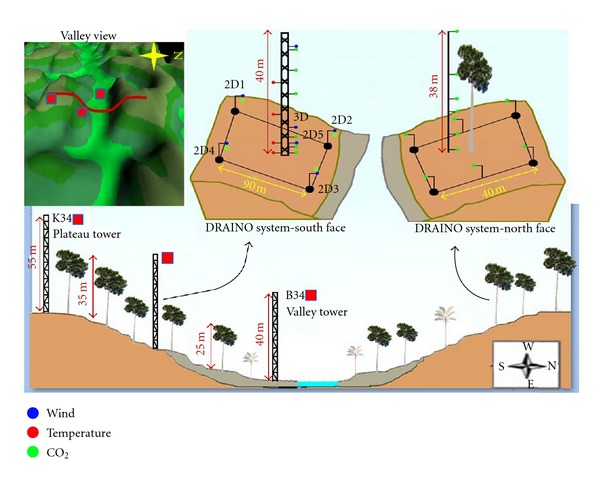
DRAINO measurement system (south and north slope face) implemented at the Manaus LBA site, including topographic view and instrumentation deployed.

The DRAINO measurement system used in the Manaus LBA site was similar to that developed by the State University of New York, under supervision of Dr. David Fitzjarrald, and applied at Santarem LBA site, including the same methodological procedures and sampling rates (see [8]). However, due to the terrain complexity, it was modified for the Manaus forest conditions including a long distance power line and duplication of CO_2_ observations for different slopes areas (Figure 4). The DRAINO measurement system used in the Manaus LBA site was mounted in an open, naturally ventilated wooden house (Figure 3).

The system and sensors were deployed (Figure 4) with measurements of air, temperature and humidity (red points), CO_2_ concentration (green points), and wind speed and direction (blue points), for both south and north faces. The observations of the 3D sonic anemometer were sampled at 10 Hz and all the other parameters (CO_2_, H_2_O, air temperature, and humidity) were sampled at 1 Hz (Figure 4).

The acquisition system developed at ASRC was employed [18]. It consists of a PC operating with Linux, an outboard Cyclades multiple serial port (CYCLOM-16YeP/DB25) collecting and merging serial data streams from all instruments in real time, the data being archived into 12-hour ASCII files. At Manaus LBA Site two systems in the both south and north valley slope faces were mounted (Figures 3 and 4).

For each slope face, a single LI-7000 Infrared Gas Analyzer (LI-COR Inc., Lincoln, NE, USA) was used. A multiposition valve (VICI Valco Instrument Co., Inc.) controlled by a CR23x Micrologger (Campbell Scientific, Inc., Logan, Utah, USA), which also monitored flow rates was also used.This procedure minimizes the potential for systematic concentration errors to obtain the horizontal and vertical profiles. Following Staebler and Fitzjarrald [17] and Tóta et al. [8] a similar field calibration was performed during the observations at the Manaus LBA site, including initial instrument intercomparison.

The result was similar to that obtained by Tóta et al. [8], with CO_2_ mean standard error < 0.05 ppm and mean standard error of about 0.005 m s^−1^ for wind speed measurements. After intercomparison, the sonic anemometers and the CO_2_ inlet tubes were deployed as shown in Figure 4.

On the south face, the instrument network array (Figure 4 and Table 1) consisted of 6 subcanopy sonic anemometers, one 3-D ATI (Applied Technologies Inc., CO, USA) at 2 m elevation in the center of the grid (named 3D ATI), and 5 SPAS/2Y (Applied Technologies Inc., CO, USA), 2-component anemometers (1 sonic at 6 m in the grid center and 4 sonic along the periphery at 2 m; see Figure 4), with a resolution of 0.01 m s^−1^. Also, a Gill HS (Gill Instruments Ltd., Lymington, UK) 3-component sonic anemometer was installed above the canopy (38 m). The horizontal gradients of CO_2_/H_2_O were measured in the array at 2 m above ground, by sampling sequentially from 4 horizontal points surrounding the main tower location at distances of 70–90 m and from points at 6 levels on the main DRAINO south face tower, performing a 3-minute cycle. On the north face, similar CO_2_ measurements were mounted including a 6 level vertical profile and 6 points in the array at 2 m above ground, performing a 3-minute cycle.

On both slope faces the air was pumped continuously through 0.9 mm Dekoron tube (Synflex 1300, Saint-Gobain Performance Plastics, Wayne, NJ, USA) tubes from meshed inlets to a manifold in a centralized box. A baseline air flow of 4 LPM from the inlets to a central manifold was maintained in all lines at all times to ensure relatively “fresh” air was being sampled. The air was pumped for 20 seconds from each inlet, across filters to limit moisture effects. The delay time for sampling was five seconds and the first 10 seconds of data were discarded. At the manifold, one line at a time was then sampled using an infrared gas analyzer (LI-7000, Licor, Inc.). To minimize instrument problems, only one LI-7000 gas analyzer sensor, for each slope face, was used to perform vertical and horizontal gradients of the CO_2_.

## 3. Results and Discussion

**Figure 5:**
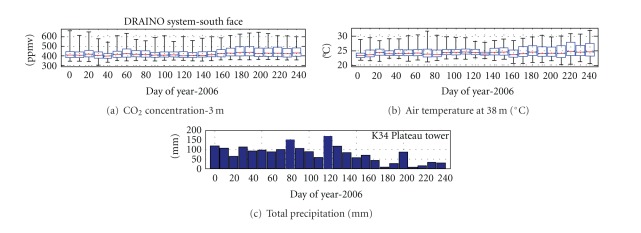
10 days time series of the CO_2_ concentration (a), air temperature (b) (DRAINO system), and total precipitation (c) (plateau tower).

The datasets analyzed in this study were obtained during the periods defined by dry (DOY 1–150 January to June) and wet (DOY 152–250 July to October) seasons of 2006.  Figure 5 presents an example of the datasets cover, with 10 days composite statistic, for CO_2_ concentration and air temperature at south face area of the DRAINO system and the total precipitation on the plateau K34 tower measurements.

The measurements covered almost the entire year of 2006, including dry, wet and the transition from wet to dry season. The air temperature amplitude above canopy on the slope area of the DRAINO system was higher, as expected, in the dry season. A good relationship is observed between CO_2_ concentration and air temperature with much large amplitudes in the dry season than in the wet season. It probably associates with less vertical mixing during dry than wet season producing much higher subcanopy CO_2_ concentration and vertical gradient along the forest.

### 3.1. Air Temperature Field

#### 3.1.1. Plateau K34 Tower

**Figure 6:**
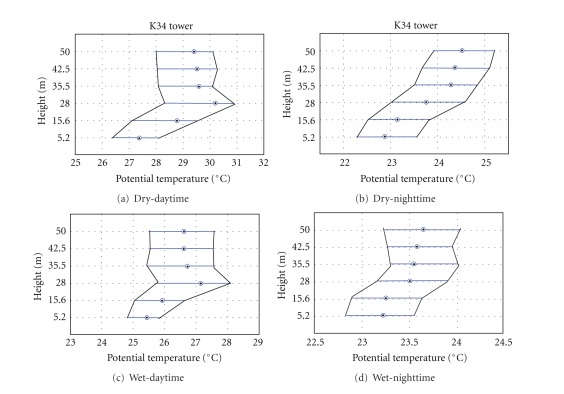
Boxplot of the virtual potential temperature vertical profile for dry ((a), (b)) and wet periods ((c), (d)) of the 2006 during night ((b), (d)) and daytime ((a), (c)), on the plateau K34 tower.

**Figure 7:**
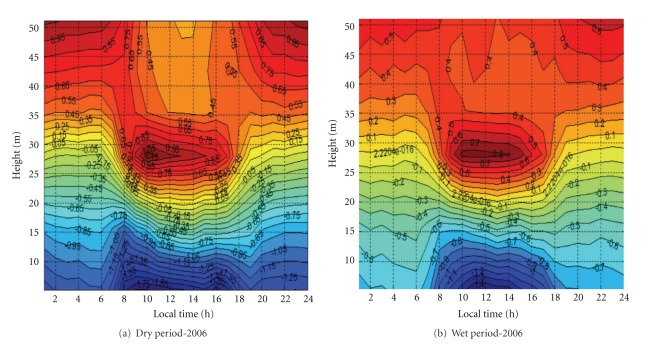
Daily course of the vertical deviation of the virtual potential temperature ([$\theta_{v}^{\prime }=\theta_{v}\left(z\right)-{\overset{®}{\theta_{v}(z)}}_{5.2}^{55}$]), during dry (a) and wet (b) periods of 2006, over plateau K34 tower.

The vertical profiles of air temperature from plateau K34 tower show a very different pattern from that on the slope area, probably due to canopy structure differences (Figure 2(b), [31]). The canopy structure is important for characterizing its thermal regime as it can be seen in Figure 6. The mean canopy layer stores large quantity of heat during the daytime and distributes it downward and upward throughout the nighttime (Figures 6 and 7).

Above the canopy layer, over plateau area, the neutral or unstable conditions were predominant during the daytime for both seasons (Figures 6(a) and 6(c)). During the nighttime, stable conditions dominate during dry period (Figure 6(b)) and neutral-to-stable conditions for the wet period (Figure 6(d)). Similar pattern has been reported elsewhere for plateau forests in the Amazonia [37–40].

The below-canopy layer of ambient air on the plateau area was stable at all times (Figures 6(a), 6(b), 6(c), 6(d)), indicating that this layer is stable where the cold air concentrated in the lower part of the canopy air space as shown in Figure 7. 

The Figure 7 presents daily course of the vertical deviation of the virtual potential temperature, for example, ([${\theta_{v}^{\prime }=\theta }_{v}\left(z\right)-{\overset{®}{\theta_{v}(z)}}_{5.2}^{55}$]), the temperature differences from each level in relation to the vertical average profile. The subcanopy air space was relatively colder during both dry and wet season, showing a similar feature of strong inversion. The same pattern was reported by Kruijt et al. [39] measured over a tower located 11 km northeast of our site with a similar forest composition.

Note that a very interesting length scale can be extracted from the observation when the deviation is about zero. The vertical length scale has mean value of about 30 m during nighttime and 20 m during daytime (yellow color in the Figures 7(a) and 7(b)). Those values are comparable with above-canopy hydrodynamic instability length scale used in most averaged wind profile models [41–44].

#### 3.1.2. DRAINO System Slope Tower

**Figure 8:**
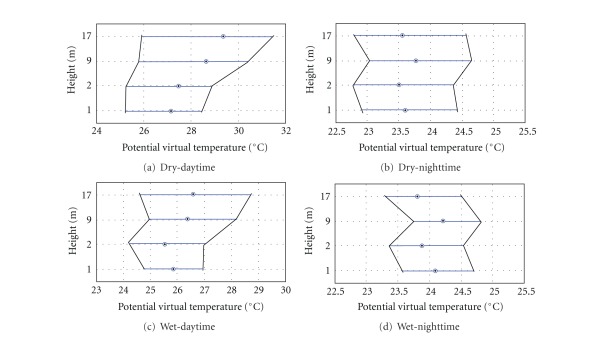
Boxplot of the virtual potential temperature vertical profile for dry ((a), (b)) and wet periods ((c), (d)) of the 2006 during night ((b), (d)) and daytime ((a), (c)), on the slope area of DRAINO System tower (south face; see Figure 2).

On the slope area south face (see Figure 2), air temperature at 5 levels underneath the canopy (heights 17, 10, 3, 2, and 1 m) was measured. The observations of the air temperature profile inside canopy are used to monitor the possible cold or warm air layer that generates drainage flow on the slope area.  Figure 8 presents observations of the virtual potential temperature vertical profile for both dry and wet periods, during both day- and nighttime. 

The pattern on the slope area is clearly very different when compared with that on the plateau K34 area (Figure 6), except in dry period during daytime when the air was stable inside the canopy. During nighttime (wet and dry periods) a very stable layer predominates with inversion at about 9 m. These can likely be interpreted as a stable layer between two convective layers is associated with cold air (Figure 8). Yi [25] hypothesized about a similar “*super stable layer*” developing during the night in sloping terrain at the Niwot Ridge AmeriFlux site. This hypothesis suggests that above this layer, vertical exchange is most important (vertical exchange zone) and below it horizontal air flow predominates (longitudinal exchange zone). The relationship between subcanopy thermal structure and the dynamic of the airflow on the slope area will be discussed in the next section.

**Figure 9:**
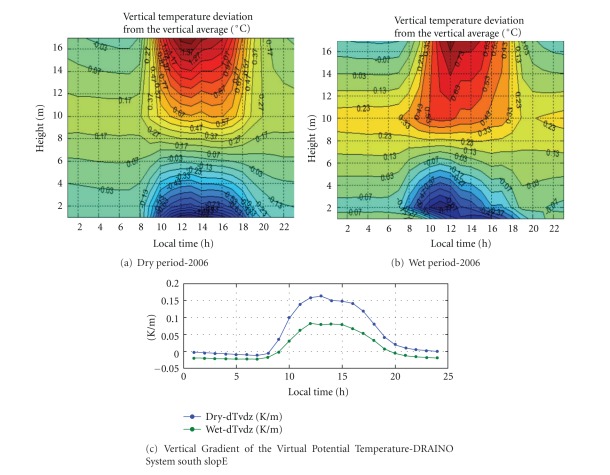
Daily course of the vertical deviation of the virtual potential temperature for dry (a) and wet (b) periods of the year 2006, and the virtual potential temperature vertical gradient (c), over slope area DRAINO system tower.

Figure 9 presents a daily cycle composite of the virtual potential temperature deviation from the vertical average ([$\theta_{v}\left(z\right)-\overset{®}{\theta_{v}}{\left(z\right)}_{1}^{18}$]). There is persistent cold air entering during nighttime for both dry and wet periods, a characteristic pattern observed on the slope area. It is a very different vertical thermal structure from that of the plateau area.

The cold air in the subcanopy upper layer is probably associated with top canopy radiative cooling, while the cold air just above floor layer is associated with upslope wind from the valley area (as discussed later in the next section).

The average of the vertical gradients virtual potential temperature was negative during nighttime and positive during daytime for both periods dry and wet (Figure 9). This observation shows that during the daytime a relative cooler subcanopy air layer predominates creating inversion conditions. In contrast, a relative hotter subcanopy air layer generates a lapse conditions during nighttime. In general that is not a classical thermal condition found on the sloping open areas without dense vegetation. This general pattern was present at several specific study cases not shown here due to limited size paper. A similar pattern was reported by Froelich and Schmid [26] during “leaf on” season.

### 3.2. Wind Field

The LBA Manaus site has moderately complex terrain when compared with the Santarem LBA site (Figures 1 and 2). This complexity generates a wind airflow regime much complex to be captured by standard measurement system like a single tower. At the Manaus LBA site, we implemented a complementary measurement system on the slope area to support the plateau K34 tower and better understand how the airflows above and below the canopy interact and also to describe how the valley flow influences the slope airflow regimes. It is important to note that the valley in the microbasin is oriented from east to west (Figures 2 and 4).

#### 3.2.1. Horizontal Wind Regime: Above Canopy

**Plateau K34 Tower.**

**Figure 10:**
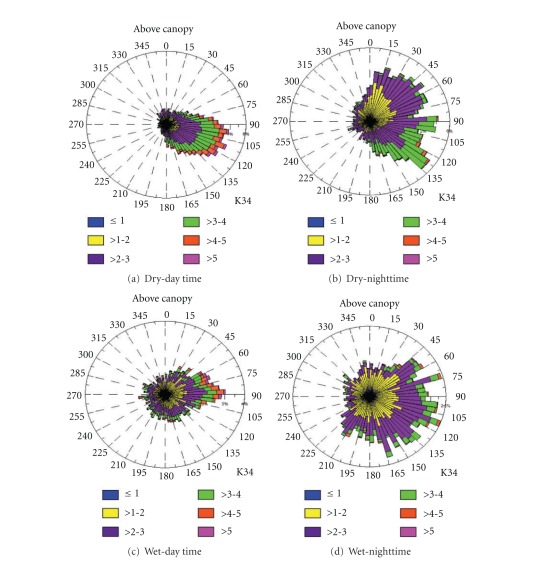
Frequency distribution of the wind speed and direction. For dry ((a), (b)) and wet ((c), (d)) periods from the year 2006 during day ((a), (c)) and nighttime ((b), (d)), on the plateau K34 tower.

Above the canopy (55 m above ground level—a.g.l.) on the plateau area of K34 tower, the wind regime was strongest (most above 2 m.s^−1^) during daytime for both dry and wet periods of 2006, with direction varying mostly from southeast and northeast for dry and wet period, respectively (Figure 10). During nighttime, the wind regime was slower (most below 3 m.s^−1^) and with same direction variation from northeast to southeast (Figure 10). As reported by de Araújo [28], the above-canopy valley area's wind speed and direction was different from that of the plateau area, suggesting a decoupling mainly during nighttime. A clear channeling effect on the valley wind regime was observed, which was oriented by microbasin topography during both day- and nighttime, with direction of the flow in the valley area determined by the valley orientation (as also reported by [28]).

**DRAINO System Slope Tower.**

**Figure 11:**
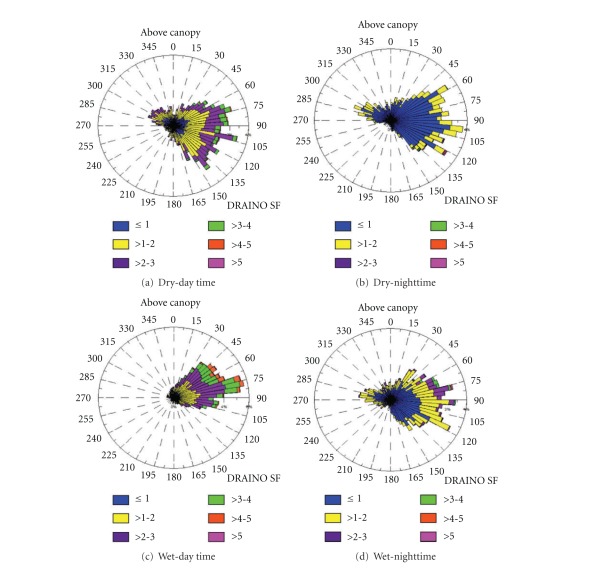
Frequency distribution of the wind speed and direction above canopy (38 m above ground level-a.g.l). For dry ((a), (b)) and wet ((c), (d)) periods from the year 2006 during day ((a), (c)) and nighttime ((b), (d)), on the slope area at DRAINO system tower.

For the above canopy (38 m above ground level—a.g.l.) on the slope area DRAINO system south face (see Figure 4, 3D sonic), the wind regime was very persistent from east quadrant direction during day- and nighttime in both dry and wet periods of the 2006 (Figure 11). The daytime wind speed during the dry season was between 1 and 3 m s^−1^ and much stronger during the wet period with values up to 4 m s^−1^.

During the nighttime the wind speed was slower than 2 m s^−1^, except from northeast during the wet period. The wind direction pattern was similar to that on the plateau K34 tower (Figure 10) prevailing from northeast to southeast. This observation indicates that the airflow above the canopy on the slope area is related to how the synoptic flow enters in the eastern part of the microbasin (see Figures 2 and 4).

#### 3.2.2. Horizontal Wind Regime: Subcanopy Array Measurements (2 m a.g.l)

**Figure 12:**
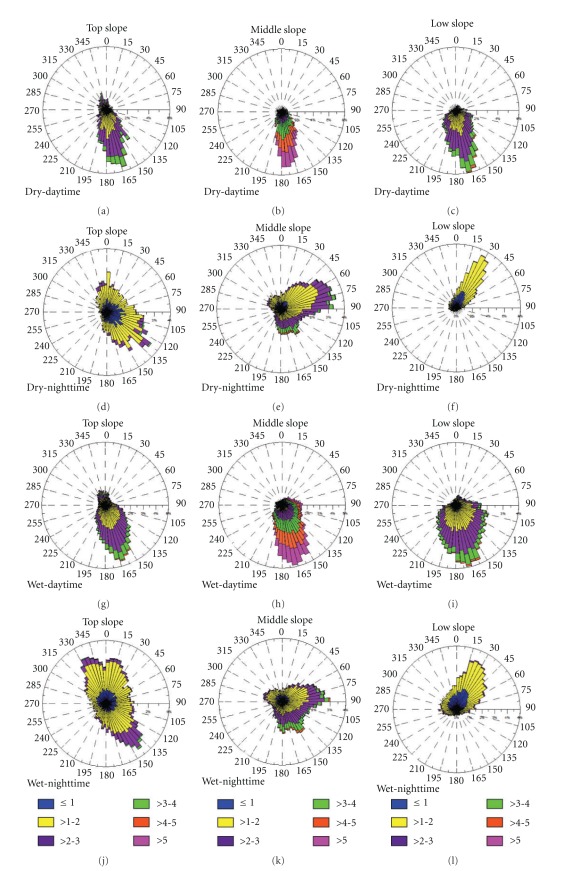
Frequency distribution of the wind speed and direction in the subcanopy array (2 m above ground level—a.g.l) on the microbasin south face slope area at DRAINO horizontal array system (see Figure 4). For dry (a–f) and wet (g–l) periods from the year 2006, during day- ((a), (b), (c), (g), (h), (i)) and nighttime ((d), (e), (f), (j), (k), (l)).

In Figure 12, the subcanopy array frequency distribution of the wind speed and directions is shown for both dry and wet periods of the year 2006, during both day- and nighttime. The observations show that the airflow in the subcanopy is very persistent and with similar pattern during both dry and wet periods of the year 2006. It is important to observe that the south slope area in the DRAINO System (see Figure 4) is *downslope* from south and *upslope* from north quadrants.

**Subcanopy Daytime Wind Regime.**

During daytime, in both dry (Figures 12(a), 12(b), and 12(c)) and wet periods (Figures 12(g), 12(h), and 12(i)), the wind direction prevailed from south-southeast (190–150 degrees) on the three slope regions (Figure 12, Top ((a), (d), (g), (j)), Middle ((b), (e), (h), (k)) and Low slope part ((c), (f), (i), (l))). The airflow in the subcanopy was decoupled from the wind regime above the canopy (Figure 11) most of the time. The wind direction in the subcanopy airflow was dominated by a daytime downslope regime during the majority of the period of study, suggesting a systematic daytime katabatic wind pattern.

The wind speed in the subcanopy during the daytime was mostly from 0.1 to 0.4 m/s, and strongest at middle slope region (Figure 12(b), 12(e), 12(h), 12(k)) about 0.3 to 0.5 m/s or above. A similar daytime katabatic wind regime was reported by Froelich and Schmid [26] during “leaf on” season in Morgan-Monroe State Forest (MMSF), Indiana USA.

The daytime downslope wind was also supported by the subcanopy thermal structure (Figure 9), where the air was cooling along the day by inversion of the virtual potential temperature profile with a positive vertical gradient (Figure 9(c)). This results shows that subcanopy flows in a sloping dense tropical rainforest are opposite to the classical diurnal patterns of slope flows studied elsewhere in the literature (e.g., [45–48]). It is important to note that few studies have been done in forested terrain and it is unclear why similar reversed diurnal patterns have not been observed in studies at other forested sites [15, 17, 49], except by a single point subcanopy measurement observed by Froelich and Schmid [26].

**Subcanopy Nighttime Wind Regime.**

The nighttime subcanopy wind regime on the slope area (see the terrain on Figure 4) was very complex and differentiates from that one above the canopy vegetation. It was observed that, on the upslope part, the nighttime airflow was southeast downsloping direction (130°−170°) and northeast-northwest (45°−340°) uphill direction (Figures 12(d) and 12(j)). In the middle-part of slope area, the wind moved uphill (from northeast; 30°−90°) and also downsloping wind direction from southeast (Figures 12(e) and 12(k)), and with lightly higher wind speed. On the lower part of the slope area (Figures 12(f) and 12(l)) the wind direction prevailed from the northeast (10°−70°), indicating upsloping pattern (anabatic). It is interesting to note that, on the up-slope area, the wind direction regime (northeast-northwest, 45°−340°) suggest a reversal lee side airflow (recirculation or separation zone) probably in response to the above canopy wind (see Figures 11(b) and 11(d)). It has been suggested by Staebler [50] and reported by simulations using fluid dynamic models [51, 52].

**Figure 13:**
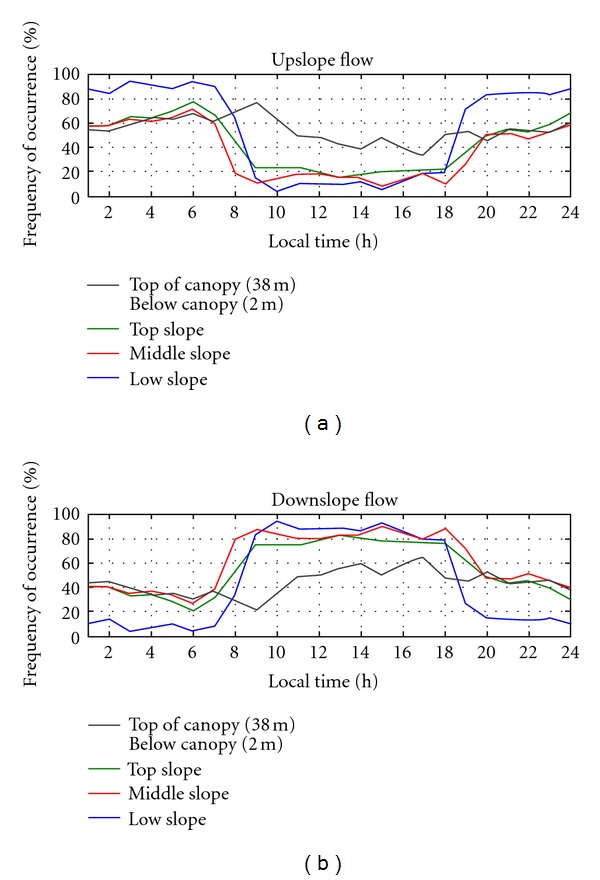
Frequency distribution of the subcanopy wind direction (a) upsloping (from north quadrant) and (b) downsloping (from south quadrant) on the south face slope area at the DRAINO horizontal array system (see Figure 4).

The upsloping subcanopy flows pattern, on the lower part of the slope area, is supported by the subcanopy's relative heat air layer along the slope during the night, as observed by lapse rate condition of the virtual potential temperature negative vertical gradient (Figure 9(c)). This observation does not follow the classical concept of nighttime slope flow pattern, as commented previously (Section 3.1.2) this is an example of nonclassical microscale slope flow. Froelich and Schmid [26], has reported similar feature where they found anabatic wind regime during nighttime in their seasonal forest study area.  Figure 13 presents the frequency distribution of the subcanopy wind direction on the south face slope area at the DRAINO horizontal array system during upsloping (from north quadrant) and downsloping (from south quadrant) events.

#### 3.2.3. Mean Vertical Wind Velocity: Subcanopy and Fbove Canopy

**Figure 14:**
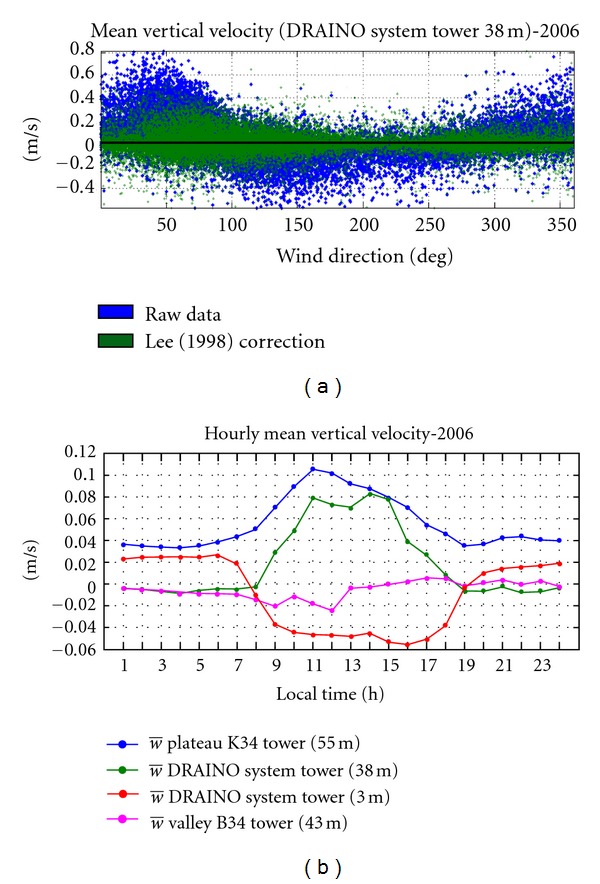
Mean vertical velocity raw and correct vertical velocity (a) for DRAINO system slope tower (38 m), and hourly mean vertical velocity (b) for: plateau K34 tower (55 m), DRAINO system slope tower (above canopy 38 m and subcanopy 3 m) and for valley B34 (43 m) towers (see Figure 4, for details).

Several correction methods have been proposed to calculate the mean vertical velocity, for example, linear regression method [12], coordinate rotation [53], and the planar fit method [54]. We use the linear regression method by Lee [12] to determine the “true” mean vertical velocity: $\overset{®}{w}=w-a\left(\alpha_{i}\right)-b\left(\alpha_{i}\right)u$, where “*a*” and “*b*” are coefficients to be determine, for each *α*_*i*_ (10° azimuthal wind direction), by a linear regression of measured mean vertical velocity (*w*) and horizontal velocity (*u*) in the instrument coordinate system.  Figure 14(a) presents the original and the correction results by method application of the mean vertical velocity as function of wind direction. In Figure 14(b), the results of the hourly mean vertical velocities for plateau K34, DRAINO system (above and below canopy), and valley B34 towers are shown. As expected, not only low but non-zero values were observed for all points of measurements as well. 

On the plateau area, the mean vertical velocity was always positive indicating upward motion or vertical convergence at the top of the hill during night- and daytime. In the valley area during nighttime, negative or zero values were observed, indicating a suppression of vertical motion (mixing) in the valley, as also reported by de Araújo [28].

However, during the daytime, a transition is observed, where beginning in the morning, downward motion is observed, changing after midmorning to upward motion (Figure 14(b)). This suggests that probably the cold air pooled during night moved downslope and started to warm, resulting in a breakdown the inversion over the valley (see [28], for detailed description and references therein for this process). The mechanism of the breakdown, the inversion process over the valley, is consistent with positive vertical velocity observed above canopy at slope area by the DRAINO system tower during daytime (Figure 14(b)).

The subcanopy diurnal pattern of the mean vertical velocity observed shows positive values during nighttime and negative during daytime, consistent with observed up- and downsloping flow regime, respectively (Figures 13(a) and 13(b)). This is consistent with thermal vertical virtual potential temperature gradient on the slope (see Figure 9(c)), where, during nighttime (daytime), an unstable (inversion) condition is associated with upward (downward) mean vertical velocity (see Figure 9(c)).

### 3.3. Phenomenology of the Local Circulations: Summary

**Figure 15:**
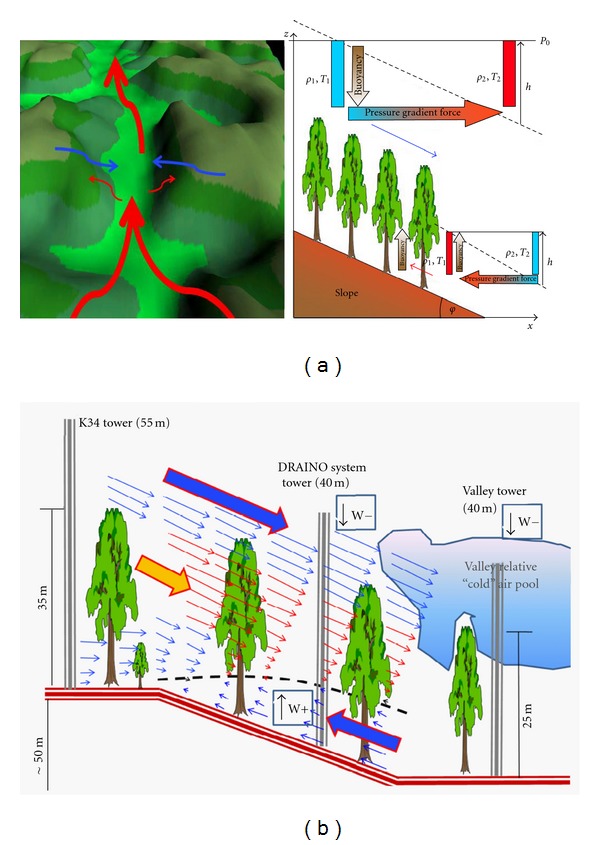
Schematic local circulations in the site studied, valley and slopes flow (a), 2D view from suggested below and above canopy airflow (b).

Figure 15 shows a schematic cartoon of local flow circulation from the previews sections observations.

In Figure 15(a) is shown the above canopy airflow over valley space (red arrow) and correspondently (induced) the most probable airflow above canopy over slope areas (blue arrow). In the same figure is shown the main physical mechanisms (pressure gradient force) producing that microscale circulations. The observations result from preview sessions suggesting that the balance of the buoyancy and pressure gradient forces generate the airflow or microcirculation patterns in the site studied.

During nighttime (Figure 15(b)), in the subcanopy, there is an upslope flow reaching about 10 m height above the ground, associated with positive mean vertical velocity (indicating upward movement). Also, above canopy, there is a downslope flow associated with negative mean vertical velocity, with downward convergence above the canopy. The microcirculation along the plateau-slope valley is promoted by a feedback mechanism of accumulation of cold air drainage above canopy into the valley center (Figure 15(b)), creating the forcing needed to sustain nighttime pattern. The air temperature structure above canopy in the valley (see [28]) is a good indication of cold air pool in the center of the valley. Maybe the local pressure gradient force due to the cold air accumulation promotes the upward airflow in both the slopes of the valley. During daytime periods, an inverse pattern is found (not show), indicating that this microcirculation is a systematic pattern in the site.

### 3.4. CO_2_ Concentration and Subcanopy Horizontal Wind Field

**Figure 16:**
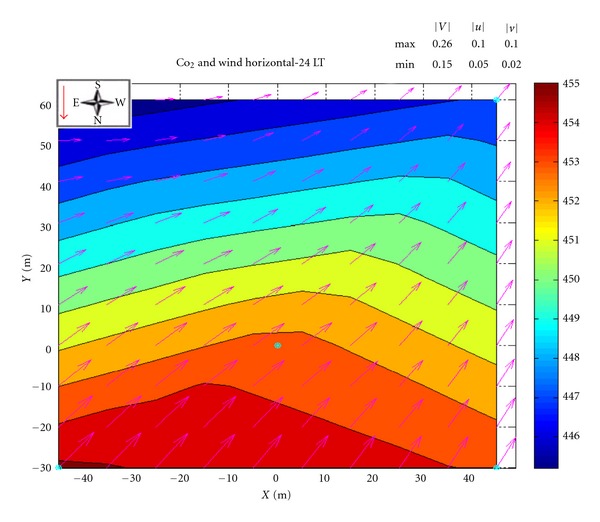
Example at midnight (local time) of the horizontal CO_2_ concentration (ppmv) over the DRAINO System south face domain including an interpolated horizontal wind field (10 m grid). Note the geographic orientation and the red arrow indicating slope inclination (see Figure 4).

The CO_2_ concentration was measured by DRAINO system on the south face slope area for dry and wet periods of the year 2006, and on the north face slope during the dry period (Figure 4). The Figure 16 presents an example, for midnight (local time), of the horizontal wind field and spatial CO_2_ concentration over the DRAINO System south face domain.

The wind field was interpolated from the blue points onto a 10 m grid. Similar procedures have been reported in the literature [19, 55]. The horizontal wind regime plays an important role in modulating the horizontal spatial distribution of CO_2_ concentration (Figure 16).

**Figure 17:**
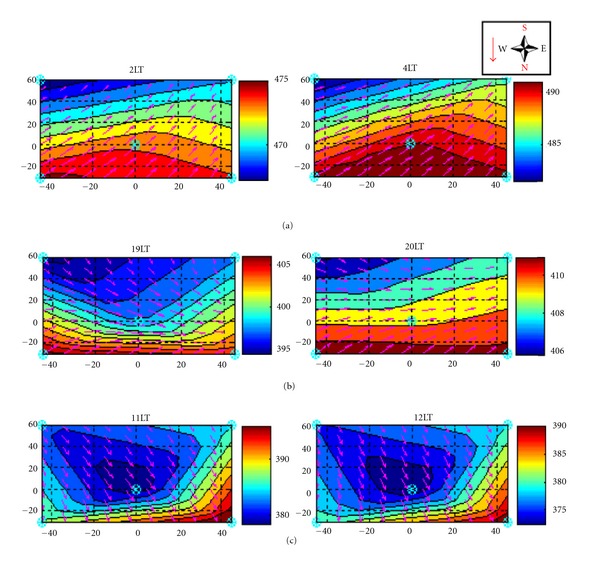
Hourly average of the subcanopy (2 m) CO_2_ concentration and horizontal wind speed over the DRAINO system south face area during dry period of the year 2006 note the geographic orientation and the red arrow indicating slope inclination (see Figure 4). The axis represents distances from center of the main tower. Daytime (a), transition period—evening (b), established nighttime (c).

In Figures 17(a), 17(b), and 17(c) the typical pattern observed is shown for both dry and wet periods of the year 2006 measured by the DRAINO system on the south-facing slope area. During the daytime (Figure 17(c)), the wind prevailed downslope inducing a strong horizontal gradient of CO_2_ in the slope area (about 0.2 ppmv m^−1^). In the evening, periods of changes of the horizontal wind pattern (as described in Section 3.1) show an upsloping regime in the lower part and downsloping in the upper part of the slope areas (Figure 17(b)). The wind regimes produce direct responses in the spatial feature of the horizontal gradient of CO_2_ concentration. Later during the night, the upsloping regime is well established and also the horizontal gradient of CO_2_ is growing from lower part of the slope to the top (Figure 17(a)).

These observations suggest a subcanopy drainage flow and its influence on the scalar spatial distribution. Therefore, as discussed in the previews' sections, the flow above the canopy indicates a reverse pattern of downward motion (negative mean vertical velocity; see Section 3.2.3) that suggests vertical convergence and possible horizontally divergent flow during nighttime. The report by Froelich and Schmid [26] and more recently Feigenwinter et al. [23, 24] describe similar features of the airflow interaction between above and below canopy.

**Figure 18:**
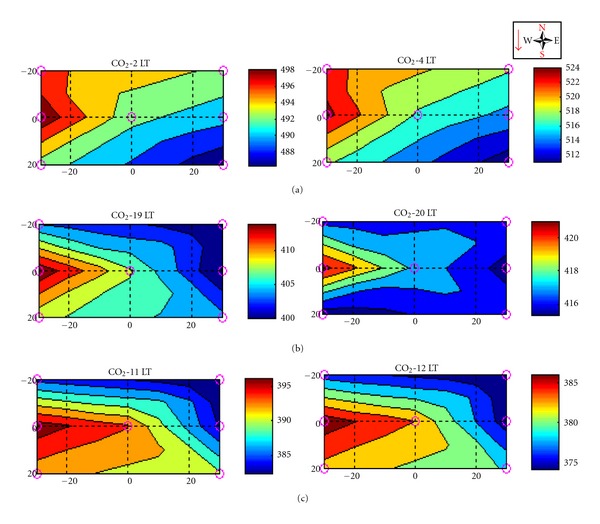
Hourly average of the subcanopy (2 m) CO_2_ concentration on the DRAINO system north face area during dry period of the year 2006. Note the geographic orientation and the red arrow indicating slope inclination (see Figure 4). The axis represents distances from center of the main tower. Daytime (a), transition period-evening (b), established nighttime (c).

The spatial distribution of the horizontal CO_2_ concentration, (Figure 18) along the north face, shows a similar pattern than the south face described previously. Despite there is no wind information in that area, if one assumes the same spatial correlation between horizontal wind and CO_2_ concentration, it is possible predict that the wind should present an inverse pattern from the south face suggesting that, during daytime, the downslope wind direction should be from the northeast (Figure 18(c), from blue to red color).

During the evening period (Figure 18(b)), it should be indicating downslope (from northeast) in the upper part of the north face slope and upslope (from southeast) in the lower part of the slope, an inverse feature from Figure 17(b). Finally, later in the night, on the north face slope, the wind pattern should present an upslope wind direction regime from southeast, an inverse regime to that one from Figure 17(a) on the south face slope.

One possible explanation to this subcanopy slopes' wind regime and spatial distribution of CO_2_ concentration, is the valley wind channeling effect and how it is meandering when it enters in the valley topography (as described by [28]). This valley wind pattern probably causes oscillations as those observed on the CO_2_ concentration along the day (Figures 17 and 18), the known “Seiche phenomena” [56].

## 4. Summary and Conclusions

The main objective of this study was to measure and understand the local circulation over a dense forest site in Manaus with moderately complex terrain and to verify the existence of the drainage flow regimes on slope and valley areas. The main pattern of the airflow above and below the canopy in dense tropical forest in Amazonia was captured by a relative simple measure system, as also has been done by more sophisticated measurement system as those described recently by Feigenwinter et al. [23, 24]. As described and discussed in preview sections, it was identified as drainage flow in both day- and nighttime periods in the site studied. Evidence of the drainage current above canopy was suggested by Goulden et al. [40] similar to the one observed here. The study highlighted that the local microcirculation was complicated and presented tridimensional nature. Where to estimate the advection flux at this site seems uncertain and not possible with the limited measurement system employed. As reported recently by Feigenwinter et al. [23, 24], even using a more sophisticated measurement design, the level of uncertainties is still high and some processes are not yet known and need more exploration perhaps using a more complete spatial observation network or even applying model resources [57, 58].

In summary, the drainage flow exists and is observed at the K34 LBA site. Very large carbon uptake estimates reported previously should be questioned [27, 29], and more research is warranted. The use of nighttime u* correction to avoid estimating canopy storage is inappropriate. One cannot get by using only the above-canopy turbulence information. The interactions between motions above and below canopy question the foundations of the footprint analysis [59, 60]. The representativeness of the eddy flux tower is most in question for complex terrain, especially on calm nights.

## Figures and Tables

**Table 1:** DRAINO system sensors at ZF2 LBA Manaus site.

Level (m)	Parameter	Instrument
38	*u*′ *v*′ *w*′ *T*′	Gill 3D sonic anemometers
2	*u*′ *v*′ *w*′ *T*′	ATI 3D sonic anemometer
6, 2	*u*′ *v*′ *w*′ *T*′	CATI/2 2D sonic anemometers
2	CO_2_ concentration (horizontal array)	LI-7000 CO_2_/H_2_O analyzer
38, 26, 15, 3, 2, 1	CO_2_, H_2_0 profile (sourth face)	LI-7000 CO_2_/H_2_O analyzer
35, 20, 15, 11, 6, 1	CO_2_, H_2_0 profile (north face)	LI-7000 CO_2_/H_2_O analyzer
18, 10, 2, 1	Air temperature and humidity	Aspirated thermocouples

*u*′ *v*′ *w*′: wind components and *T*′: air temperature fluctuation.
